# Repetitive negative thinking moderates the association between self-referential thoughts and cognition amongst older adults: a natural speech analysis approach

**DOI:** 10.21203/rs.3.rs-10775960/v1

**Published:** 2026-09-04

**Authors:** Cristina Solé-Padullés, Oriol Perera-Cruz, Arnau Güell-Brugués, María Cabello-Toscano, Lídia Mulet-Pons, Rachel M. Morse, Gabriele Cattaneo, Javier Solana-Sánchez, Natalie L. Marchant, Álvaro Pascual-Leone, David Bartrés-Faz

**Affiliations:** University of Barcelona; University of Barcelona; University of Barcelona; University of Barcelona; University of Barcelona; University of Barcelona; Institut Guttmann; Institut Guttmann; University College London; Hinda and Arthur Marcus Institute for Aging Research; University of Barcelona

**Keywords:** think aloud paradigm, repetitive negative thinking, episodic memory, executive functions, cognitive resilience

## Abstract

Natural language processing has been examined as a possible biomarker for cognitive decline and dementia, but it has also been associated with components of well-being and psychological distress. Additionally, there are psychological variables that may influence contents of thoughts during spontaneous speech. We aimed to explore whether self-referential thoughts, emotional tone and time orientation, measured during a think aloud paradigm and analyzed using natural language processing, were related to cognitive performance in older adults, and whether psychological characteristics influenced these associations. In total, 110 cognitively healthy adults aged 65 years and above were recruited. Cognitive assessment included tests of episodic memory and executive functions, and participants completed psychological questionnaires of depressive and anxiety symptoms, personality traits, repetitive negative thinking, and purpose in life. Increased proportion of self-related words, used as a proxy for self-referential thoughts, was associated with poorer episodic memory and executive functions. These associations were moderated by levels of rumination and worry, respectively, two components of repetitive negative thinking reflecting cognitive debt and previously related to reduced cognitive resilience. We concluded that self-referential thoughts measured through natural speech represent an ecological marker related to suboptimal cognitive performance when linked to high levels of repetitive negative thinking, even beyond the influence of depressive and anxiety symptoms, and we highlight the need to consider psychological factors when addressing natural speech studies with a focus on aging and cognition.

## Introduction

Cognitive functions are traditionally assessed through validated neuropsychological tests and customized protocols to explore cognitive domains [1]. Within the last decade, several computerized tests have emerged as efficient tools to evaluate larger samples in a cost and time-efficient manner [2–6]. More recently, natural language patterns have also been introduced as a possible biomarker for cognitive decline and dementia [7]. In a recent review which included 79 studies considering both linguistic and non-linguistic parameters, the authors concluded that natural language processing (NLP) can distinguish between different cognitive stages, monitor Alzheimer’s disease (AD) patients, as well as evaluate the associated social burden [8]. Accordingly, most NLP studies have focused on comparisons between clinical populations and healthy controls. However, a recent study went a step further by accurately classifying a continuum of cognitive stages, from normal to impaired cognition, using innovative NLP methods that analyze semantic meaning [9].

Beyond clinical applications, NLP has also been used to explore more psychological aspects of healthy aging, such as different components of well-being, including affective, evaluative and social features [10]. Nevertheless, to our knowledge, no studies have specifically attempted to examine the relationship between emotional characteristics of spontaneous speech and cognitive performance in healthy aging, particularly in higher-order cognitive functions that are more prone to decline with age, such as episodic memory and executive functions. Additionally, further investigation is needed to determine whether psychological factors associated with dementia risk may also play a role in the putative associations between spontaneous language production and cognition.

In this line, and beyond the acknowledged risk and protective factors for dementia documented in the past Lancet commission [11], recent investigations point to the relevance of processes of a more psychological nature [12], which could act as risk factors to accelerate cognitive decline or as protective factors to promote resilience. For instance, Repetitive Negative Thinking (RNT), a cognitive process characterized by ruminative brooding and worry, which can lead to emotional distress and anxiety-related disorders [13], has also been associated with increased risk for subjective and objective cognitive decline as well as with the presence of AD-related biomarkers [14–16].

Key components of RNT, namely ruminative brooding and worry, are acknowledged as thinking styles that could likely be reflected in natural speech. In this line, in an analysis of natural language using the Think Aloud Paradigm (TAP) with young adults, Raffaelli and colleagues [17] found that ruminative brooding was characterized by more negative, past-oriented and self-referential thoughts. However, the authors did not examine possible associations between natural language production and cognition, as the age range of the sample did not correspond to a population at a higher risk of cognitive decline.

Additionally, personality traits have also been identified as psychological characteristics associated with cognitive trajectories during aging. In particular, neuroticism has been linked to an increased risk of progression to mild cognitive impairment, whereas conscientiousness appears to confer some protection [18]. Other personality traits, including openness to experience, extraversion and agreeableness, have also been related to lower risk of dementia [19]. Beyond personality, other psychological protective factors have been linked to cognitive health in middle aged and later life, including self-reflection and purpose in life [12]. Self-reflection has been associated with better global cognition in independent cohorts of cognitively unimpaired older adults and individuals with subjective cognitive decline [20]. Similarly, higher purpose in life has been related to greater functional connectivity in target memory-related brain regions, which in turn has been associated with better cognitive performance [21].

In the present study, we aimed to examine whether specific TAP-derived measures, namely percentage of self-referential speech, emotional tone and time orientation, were associated with cognitive performance in healthy older adults. We also investigated whether these associations, if any, varied according to psychological characteristics previously defined as aggregating into psychological profiles of risk or protection for dementia [12]; specifically, RNT components (brooding and worry), personality traits, purpose in life (PiL) and self-reflection.

## Method

### Participants

A total of 110 participants from a follow-up cohort of healthy adults aged > 65 years completed the TAP and in-person cognitive assessments (see below) in Barcelona, Spain. Exclusion criteria included previous diagnosis of any psychiatric or neurological disease, significant brain injury, Mini-Mental State Examination score lower than 25, or severe visual or auditory impairments. Psychological questionnaires to evaluate personality, RNT and Purpose in Life (PiL) were also administered, and full scores were available for 108 participants. All study procedures followed the guidelines and regulations approved by the Ethics Committee from the University of Barcelona (February, 2024, Institutional Review Board IRB00003099) and written informed consent was obtained for all volunteers.

### Think Aloud Paradigm (TAP)

Participants were audio-recorded while sitting and voicing aloud their thoughts alone in a neutral testing room for 5 minutes. As in Raffaelli and colleagues [17], they were asked to concentrate on their present thoughts or whatever came to their minds, whether it was related to internal or external stimuli, without any censorship and without standing up from their chair. No other prompts were given. Being Catalonia a bilingual society, with both Catalan and Spanish as official languages, we asked participants to talk in the language of their preference. Thus, 65.46% of participants chose Catalan and the remaining 34.56%, Spanish. Immediately after the 5-minute TAP task, participants offered feedback regarding how similar their thoughts had been from those that they usually had in their daily life (‘0’ being not similar at all to ‘1’ being very similar) as well as the degree of censorship during the task (‘0’ none at all to ‘1’, a maximum censorship).

### TAP analysis: Linguistic Inquiry and Word Count

All TAP audio-recordings were automatically transcribed using the Whisper medium speech-to-text model [22], and later manually reviewed by two investigators bilingual in Catalan/Spanish. To comply with privacy requirements, the automatic transcription was run locally and sensitive data were removed. The software to analyze Linguistic Inquiry and Word Count (LIWC-22) software was used to analyze patterns of language [23], which provides a full dictionary in English. Therefore, and as recommended by its creators, all transcripts were translated into this language. To do so, we used the LLM model GPT-4o [24]. The data for LLM training were not shared, and a prompt was used to preserve the original style as much as possible. Then, the translated texts were reviewed by an investigator fluent in English, Spanish and Catalan to ensure they perfectly matched the original transcripts. Once the translated texts were ready, the LIWC-22 software was run, computing the percentage of self-pronouns as a proxy of self-referential thoughts, as well as parameters reflecting emotional tone and time-orientation (see below).

### Cognitive assessment

A complete neuropsychological battery was administered immediately after the TAP task with tests of verbal episodic memory and executive functions, namely they Rey-Auditory Verbal Learning Test (RAVLT) without the interference list, as well as Trail Making Test A and B, Symbol Digit Modalities Test (SDMT) and Digit span (forward and backwards) from WAIS-IV. For episodic memory, a composite score was created by averaging the Z scores for total words learnt during the five trials and the delayed recall score 20 minutes after the final learning trial. Likewise, a composite score of executive functions was obtained following the same procedure and comprising the inverse ratio of Trail Making Test B/A, SDMT correct items and Digit spans. Composite scores were created following similar procedures as in a previous study [25].

### Psychological questionnaires

Following a previous investigation by our group, which identified associations between psychological characteristics and cognition as well as brain status [12], we obtained the same psychological characteristics through the following self-administered questionnaires: ruminative brooding and self-reflection (extracted from the Rumination Responses Scale; [26]), worry (Penn-state worry questionnaire-short, [27]), Purpose in Life (PiL, subscale of the Psychological Well-being Scale, [28]), and the International Personality Item Pool [29]. Thus, we obtained five personality traits: emotional stability, conscientiousness, openness to experience, extraversion and agreeableness. Unlike our earlier investigation, we did not aim to obtain psychological profiles but rather sought to examine which of the abovementioned psychological characteristics might moderate the putative associations between cognition and TAP-derived parameters. To control for depressive symptoms in our analyses, we also obtained the score in the DASS-Depression, Anxiety and Stress Scale-21 (DASS-21; [30]).

### Statistical Analysis

To explore potential links between cognition and parameters of natural language, multiple regression analyses were conducted, using episodic memory and executive functions as the dependent variables in each model. Independent variables included demographics (age, sex and years of education) and 9 TAP features reflecting the three targeted categories of interest (self-reference, time orientation and emotional tone), derived from the LIWC-22. The targeted features were: 1) The percentage of self-personal words, such as ‘I, me, my, myself’, which here we interpreted as a proxy of ‘self-referential thoughts’. For time orientation variables, we obtained the: 2) focus-past and 3) focus-future categories, which provided a percentage of past and future-related words, respectively, both including adverbs of time and verb conjugations. 4) A score indicating ‘emotional tone’ was computed from the relative use of negative and positive emotion words and then reported as a percentile score ranging from 1 to 99. Higher scores reflected a more positive tone. Other categories associated with positive or negative affect were percentage of: 5) emotion-positive, 6) emotion-negative, 7) emotion-anger, 8) emotion-anxiety and 9) emotion-sad-related words. We used a stepwise approach and only variables showing an alpha-to-enter significance level lower than 0.05 were retained. Confidence intervals for unstandardized coefficients were set to 95%. Data were analyzed using IBM SPSS Statistics (Version 29.0) and JASP (Version 0.19.3, JASP Team, 2025).

Further, moderation analyses were conducted to explore if possible associations between TAP measures and cognition were constant or rather dependent on a third variable. Using a hypothesis-driven approach, we took into account the psychological measures listed above as possible moderators; namely, PiL, self-reflection, ruminative brooding, worry, as well as the five personality traits: emotional stability, conscientiousness, openness, extraversion and agreeableness. To run the moderation analysis, we first created standardized values for all variables, including the moderators, as recommended elsewhere [31]. Then, we calculated the interaction terms (intercept) by multiplying z-scores for the targeted TAP variable and the alleged moderators. As a result, 9 interaction terms were computed, one for each psychological characteristic. Finally, we ran two unique linear regression models; one for each predicted variable (episodic memory and executive functions). For the predictors, the following terms were included: age, sex, years of education, the interaction terms and the TAP variable in absolute values. Prior to this final step, we conducted a collinearity analysis across our predictors and interactions to check the Variance Inflation factor (VIF) and determine whether it was more advisable to run one model for each interaction term or a unique model with all [32]. As suggested, when the VIF value is above 5, the collinearity is considered high, and separate regression models are hence recommended. Our collinearity diagnosis resulted in values ranging 1.13 to 2.31. With collinearity thus controlled, we ran two single regression models, one for each predicted variable. This unique approach was preferable, as it allows examination of which interactions are significant while controlling for other interactions. Homoscedasticity was evaluated through visual inspection of residuals versus predicted values, showing no apparent heteroscedasticity. Autocorrelation was assessed using the Durbin-Watson statistic (DW = 1.94 for the episodic memory model and DW = 1.95 for the executive functions model), suggesting independence of residuals. Normality of residuals was examined via Q-Q plots.

The models were carried out with and without the DASS-21 questionnaire score, to control for depressive symptoms.

## Results

The age range for the total analyzed sample was 66 to 84 years (mean age 73.94 years), 65.46% female, with a mean of years of education of 14.29, and a mean MMSE score of 28.74 (range between 25 and 30).

Participants reported that speech during TAP was highly similar to their daily natural trains of thoughts (mean similarity = 0.82, SD = 0.02), and that they did not significantly censure themselves (mean censorship = 0.09, SD = 0.17). The average word count during the 5-min TAP task was 576.94 (SD = 122.58). Linguistic authenticity was assessed using the LIWC Authenticity dimension, which provides a percentile score based on previously exploration of linguistic styles [33]. The degree of authenticity in our sample was within the 90th percentile, indicating that speech production was genuine for all participants.

Stepwise linear regression models to establish a possible association between cognition and TAP variables revealed that, for episodic memory, factors retained in order were: sex and self-referential thoughts. More specifically, female participants performed better than males (Unstandardized beta (*b*) = 0.79, Standard Error (SE) = 0.20; *t* = 4.02, *p* < 0.001; CI 95%: 0.40 to 1.18) and the higher use of selfreferential thoughts was associated with worse memory *(b*= −0.10, SE = 0.03; *t* = −3.40, *p* < 0.001; CI 95%: −0.16 to −0.04, Fig. 1A). The model explained 15.3% of the variance of episodic memory (adjusted R^2^=0.137, F = 9.65, p < 0.001).

As for executive functions, the model only retained self-referential thoughts (*b*= −0.09, SE = 0.02; *t* = −4.30, *p* < 0.001; CI 95%: −0.13 to −0.05), again pointing to a significant negative correlation between performance in tests of executive function and the percentage of self-referential thoughts during the TAP task. The model explained 14.70% of the variance of executive functions (see Fig. 1B. Adjusted R^2^ = 0.14, F = 18.51, p < 0.001).

In line with the second objective of the present study, we conducted two moderation analyses to determine whether the previously described associations between cognition and the TAP-derived variable of self-referential thoughts were moderated by the targeted psychological variables: PiL, self-reflection, brooding, worry and the big five personality traits.

As stated above, there was no evidence of problematic multicollinearity among predictors, as VIF values were below 3. Therefore, regression models were estimated including interaction terms and demographic covariates. Two significant moderation effects emerged: ruminative brooding moderated the association between episodic memory and self-referential thoughts (Table 1), whereas worry moderated the association between executive functions and self-referential thoughts (Table 2.). Importantly, both moderation effects remained significant after additionally controlling for depressive symptoms: Episodic memory ~ z-self-referential thoughts * z-brooding; *b*= −0.27, SE = 0.12; *t* = −2.75, *p* = 0.03; CI 95%: −0.51 to −0.026; and executive functions ~ z-self-referential thoughts * z-worry; *b*= −0.22, SE = 0.10; *t* = −2.12, *p* = 0.037; CI 95%: −0.43 to −0.01.)

Given the complexity of interpreting moderation effects, a graphical support was considered to better understand how the moderators worked. Specifically, we used a median split to divide the sample into high and low levels of the significant moderators (brooding and worry), following previous recommendations [34]. In Fig. 2, the two regression lines illustrate the relationship between self-referential thoughts and cognitive outcomes (episodic memory or executive functions) for participants with low and high levels of the respective moderators.

Low and high brooding groups were comparable in terms of percentage of self-referential thoughts: mean low brooding = 8.68 (SD = 3.07) versus mean high brooding = 9.18 (SD = 3.27), F = 0.46, p = 0.50. Likewise, low and high worry groups did not differ in the percentage of self-referential thoughts: mean low worry = 8.30 (SD = 3.02), mean high worry = 9.54 (SD = 3.21), F = 2.08, p = 0.15. Emotional tone, also derived from the LIWC-22 software, was comparable among groups, with a general positive tone, as mean scores were higher than 50: for low and high brooding groups: 65.78 (SD = 21.77) vs. 65.88 (SD = 21.69), respectively, F = 0.006, p = 0.94; for low and high worry groups: 65.88 (SD = 21.70) vs. 65.77 (SD = 21.76), respectively, F = 0.03, p = 0.86).

Complementary analyses revealed mild correlations between self-referential thoughts and some psychological variables, specifically openness to experience (r= −0.23, p = 0.017), PiL (r= −0.22, p = 0.02), and emotional stability (r= −0.19, p = 0.047). However, these associations did not remain significant after Bonferroni correction.

## Discussion

In the present study, we found significant associations between cognitive domains vulnerable to aging and a proxy of self-referential thoughts, as measured by natural speech production among unimpaired older adults. Specifically, the higher the proportion of words referred to oneself when thinking aloud, the worse the performance on both episodic memory and executive function tests.

This relationship however depended on two variables, namely ruminative brooding and worry, both of which characterize the psychological construct of Repetitive Negative Thinking (RNT). Thus, brooding moderated the association between episodic memory and self-referential thoughts, while worry moderated the association between executive functions and self-referential thoughts. More specifically, only higher levels of brooding and worry supported the associations, which remained significant after controlling for depressive and anxiety symptoms.

RNT has become the focus of recent studies examining risk factors for cognitive decline in older adults [35], and increasing evidence supports the link between RNT and subjective and objective cognitive decline [14–16, 36]. RNT is a cognitive process characterized by thinking about personal experiences in a non-productive, negative and intrusive way. This may interfere with problem solving and lead to distorted processing [37, 38], likely contributing to psychological distress and even to the development and maintenance of distinct psychopathological conditions. Consequently, RNT may constitute a key mechanism underlying the co-occurrence of depression and anxiety symptoms [39].

Given that RNT is inherently a self-referential process, its moderating role in the association between the proportion of self-referential verbalized thoughts and cognition is theoretically plausible. Such moderating effect may be explained by the influence of RNT on cognitive control processes and large-scale brain networks involved in self-referential thinking. Although theoretical frameworks point to the role of RNT as a way to keep individuals alert, allowing a certain balance between our goals and motivations [13], excessive RNT may be associated with a deficit of cognitive control, leading to difficulties disengaging from negative thoughts [37]. Poor cognitive control is thought to reflect insufficient top-down regulatory strategies, causing sustained focus on negative content. This tendency is thought to be related to self-referential experiences and deficits in working memory systems [40]. In line with this, in a previous study by our group, we demonstrated that higher levels of RNT were linked to increased activation of the executive control network and decreased activation of the salience network. These findings were accompanied by a distinct connectivity of these networks with the default mode network (DMN), suggesting an imbalance among higher-order connectivity networks that may contribute to diminish cognitive control at the expense of increasing self-referential and emotional processes [41]. While we did not examine functional connectivity in the present study, our results showed that a higher proportion of self-referential thoughts expressed in spontaneous speech was associated with lower performance in tasks measuring cognitive control, but also in tests of verbal episodic memory, which were administered immediately after the TAP. Additionally, and because these associations were moderated by processes underpinning RNT (worry and brooding), our findings suggest that self-referential thoughts are not inherently detrimental but may become problematic when accompanied by a style of cognitive processing characterized by repetitive negative representations of oneself or one’s actions. This would be in line with the Psychological Debt hypothesis (previous Cognitive Debt) which posits that accumulated psychological risk factors such as stress, depression and anxiety, but also RNT as a transdiagnostic process, increase individuals’ vulnerability to cognitive decline and reducing resilience [42, 35].

Notably, the associations encountered in the present study were significant after accounting for depressive and anxiety-related symptomatology, suggesting that RNT may contribute to cognitive functioning beyond the influence of current affective symptomatology, at least in this non-clinical sample of older adults. Such findings are in accordance with recent evidence of cognitive and brain atrophy changes associated with psychological factors even in the absence of anxiety and depression [12].

The absence of moderating effects of other potentially relevant psychological factors, such as personality traits and PiL may indicate that the associations observed were more strongly related to dynamic cognitive processes than to relatively stable individual characteristics. Personality traits represent enduring dispositions, whereas RNT reflects a style of processing referring more to *how we think about certain situations*, instead of *who we are*. Likewise, although PiL has been previously associated with greater brain resilience [21], it represents a future and goal-oriented factor linked to well-being rather than a cognitive process directly involved in ongoing thought generation.

Interestingly, self-reflection, considered the more adaptive component of rumination, also failed to moderate the association between cognition and self-referential thoughts. Although self-reflection involves self-focused thinking, it differs from brooding and worry in that it is a typically deliberate, goal-directed and active cognitive process promoting self-understanding, self-regulation and personal growth. By contrast, brooding and worry are characterized by intrusive and relatively uncontrollable negative thoughts. This distinction may explain why the latter, and not self-reflection, emerged as relevant moderators in the association between cognitive performance and self-referential thoughts in a think aloud task.

Interestingly, and as mentioned above, the fact that both groups of high and low levels of brooding and worry exhibited similar levels of self-referential thoughts suggests that self-focused speech is not inherently problematic. Therefore, the assumption that RNT processing is more likely to elicit self-oriented trains of thoughts is not necessarily true. Rather, the comparable percentage of self-referential thoughts among groups indicates that when self-focused thoughts are paired with repetitiveness, they may become maladaptive and therefore more likely to interfere with goal-directed resources required to complete cognitive tasks as effectively as possible.

Because RNT is a common symptom across depression and anxiety disorders, it represents a target for clinical interventions [43]. Mindfulness, for instance, can transiently reduce maladaptive ruminative thoughts through refocusing attention [44]. Interestingly, fMRI studies indicate that the neural correlates of mindfulness involve attention, inhibition, emotional networks, as well as the DMN [45], mirroring the same networks that we found to be desynchronized with RNT [41]. Interventions using cognitive behavioral therapy have obtained moderate effects [46, 47], which suggests that treatments should be more specialized and targeting relevant RNT constructs such as rumination-focused or worry-focused therapies. Along this line, while we know that the brain can be trained to self-regulate negative emotions [48], specific training in working memory systems has also been shown to reduce episodes of RNT [40].

Our work relates to previous studies demonstrating that the content and quality of spontaneous thoughts can be captured in a controlled ecological context through the TAP [17, 49]. However, there are some limitations that should be considered. First, our analyses were restricted to LIWC-22 features associated with ruminative brooding, namely time orientation, self-referential thoughts and emotional tone in a previous study [17]. Consequently, other linguistic dimensions potentially relevant to cognitive functioning, such as social or perceptual characteristics, were not examined. Second, our measure of “self-referential thoughts” was in fact a proportion of self-related words produced during the TAP, and should therefore be considered a proxy measure of self-referential thinking rather than a direct assessment of this construct. Finally, although transcripts were translated into English for linguistic analyses, a formal back-translation procedure was not conducted. To minimize potential translation bias, the translated versions were systematically compared with the original transcripts to ensure accuracy and preserve the natural meaning of participants’ speech.

In conclusion, self-referential thoughts extracted via natural language production provide an ecological and sensitive measure that appears to be associated with cognitive functioning in non-clinical older adults. However, the cognitive disadvantages were only seen when self-referential thoughts co-occurred with a maladaptive thinking style characterized by elevated brooding and worry. This finding supports the notion that the cognitive outcomes linked to self-referential processing may depend on the broader psychological context in which such thoughts occur.

In view of our findings, future research examining cognition and employing natural speech processing methodologies should consider psychological risk factors, such as components of RNT, particularly among individuals at risk of dementia. In addition, advances in automated natural language analysis could incorporate richer semantic and contextual features of language, allowing for a more nuanced characterization of thought content. Finally, future studies should examine whether interventions targeting specific characteristics of spontaneous trains of thoughts, including for instance their focus (self- *versus* other-oriented), may promote not only more well-being, but also more optimal use of cognitive resources.

## Figures and Tables

**Figure 1:**
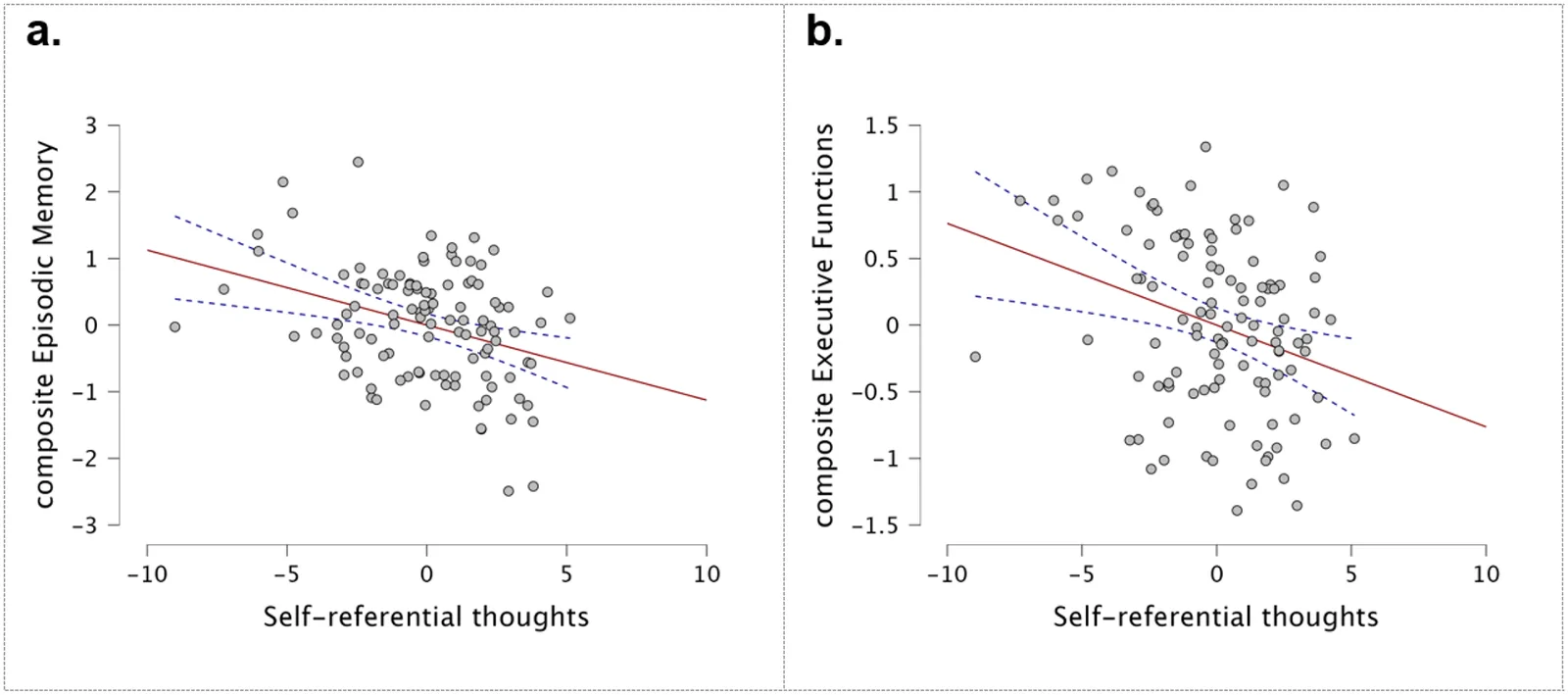
Partial regression plots depicting negative associations between self-referential thoughts and a composite of Episodic Memory (a.) and Executive Functions (b.). Dashed lines indicate confidence intervals at 95%.

**Figure 2:**
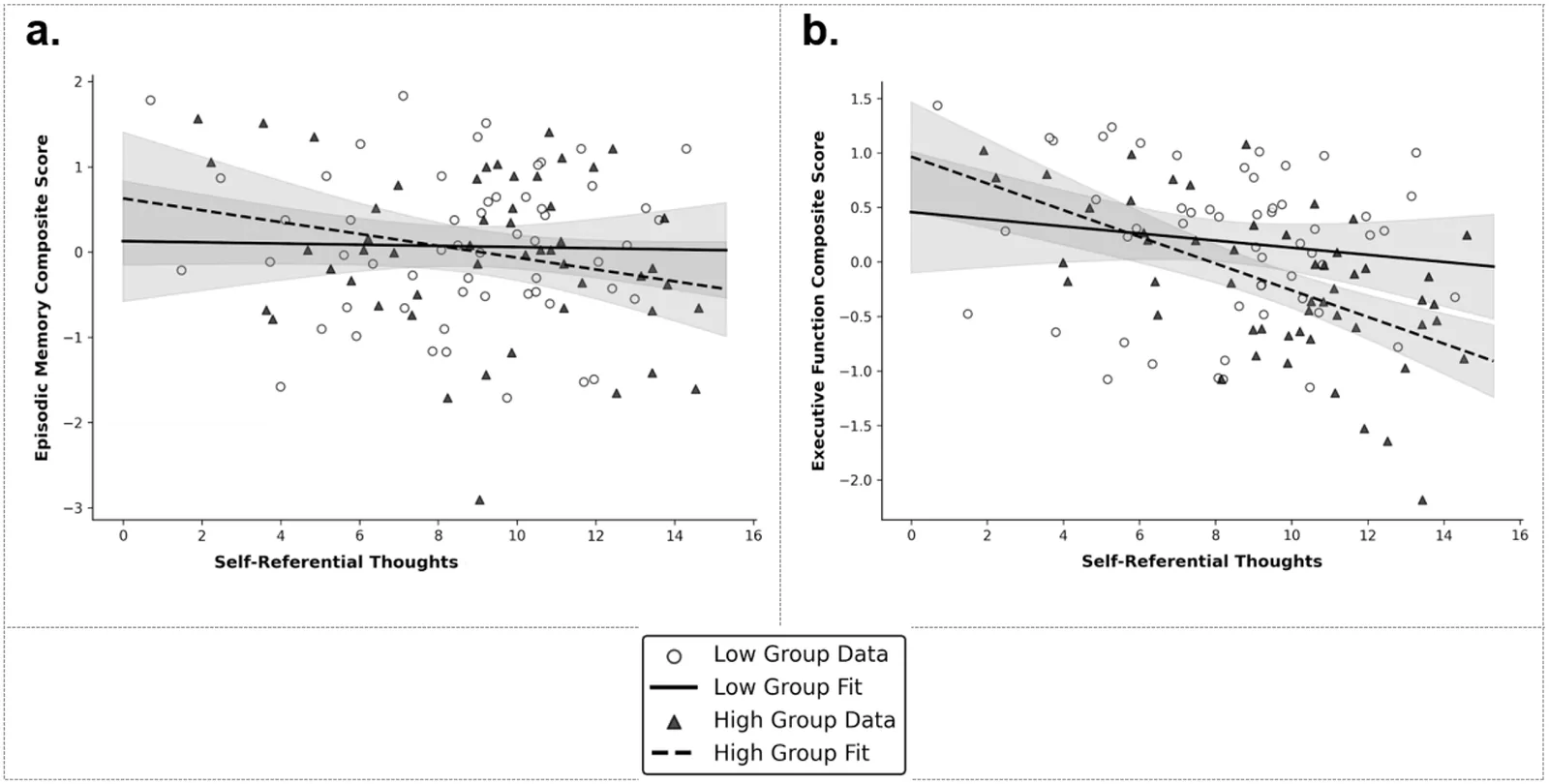
Negative association between self-referential thoughts and episodic memory moderated by brooding (a.), with the association only present within the high brooding group (dashed line, triangles), as opposed to the low brooding group (solid line, circles). On the right, the association between executive functions and self-referential thoughts as moderated by worry (b.), indicating that the association was only significant for the high worry group (dashed line). Shaded areas represent 95% confidence intervals.

**Table 1:** Moderation analysis with all interaction terms (self-referential thoughts * each psychological variable), and Episodic Memory as the predicted variable.

Model predicted variable:	Unstandardized beta	Standard Error	t value	p value	95% CI	
Episodic Memory					Lower	Upper
(Intercept)	0.798	1.657	0.482	0.631	−2.492	4.089
Age	−0.014	0.020	−0.691	0.491	−0.053	0.026
Sex (female)	0.668	0.209	3.190	0.002*	0.252	1.084
YOE	0.020	0.028	0.734	0.465	−0.035	0.076
Self-referential thoughts *Emotional stability	−0.158	0.131	−1.213	0.228	−0.418	0.101
Self-referential thoughts * PiL	0.014	0.109	0.125	0.900	−0.203	0.230
Self-referential thoughts * Brooding	**−0.309**	**0.112**	**−2.752**	0.007*	**−0.532**	**−0.086**
Self-referential thoughts * Worry	−0.038	0.133	−0.283	0.778	−0.302	0.226
Self-referential thoughts * Self-reflection	0.102	0.106	0.965	0.337	−0.108	0.312
Self-referential thoughts * Agreeableness	−0.207	0.114	−1.809	0.074	−0.434	0.020
Self-referential thoughts * Openness	0.0007	0.128	−0.005	0.996	−0.255	0.254
Self-referential thoughts * Conscientiousness	−0.055	0.105	−0.518	0.606	−0.264	0.155
Self-referential thoughts * Extraversion	0.002	0.104	0.017	0.987	−0.205	0.209
Self-referential thoughts	−0.059	0.031	−1.926	0.057	−0.121	0.002

*Note*. YOE: years of education; PiL: purpose in life; CI, Confidence interval.

**Table 2:** Moderation analysis with all interaction terms (self-referential thoughts * each psychological variable), and Executive Functions as the predicted variable.

Model predicted variable:	Unstandardized beta	Standard Error	t value	p value	95% CI	
Executive functions					Lower	Upper
(Intercept)	0.755	1.251	0.604	0.548	−1.729	3.240
Age	−0.008	0.015	−0.535	0.594	−0.038	0.022
Sex (female)	−0.038	0.159	−0.242	0.809	−0.353	0.277
YOE	0.037	0.022	1.663	0.100	−0.007	0.080
Self-referential thoughts * Emotional stability	−0.125	0.099	−1.263	0.210	−0.321	0.071
Self-referential thoughts * PiL	0.029	0.082	0.346	0.730	−0.135	0.192
Self-referential thoughts * Brooding	−0.104	0.090	−1.157	0.250	−0.284	0.075
Self-referential thoughts * Worry	**−0.215**	**0.104**	**−2.072**	0.041*	**−0.422**	**−0.009**
Self-referential thoughts * Self-reflection	−0.038	0.080	−0.475	0.636	−0.197	0.121
Self-referential thoughts * Agreeableness	−0.047	0.090	−0.520	0.605	−0.226	0.132
Self-referential thoughts * Openness	−0.113	0.097	−1.165	0.247	−0.306	0.080
Self-referential thoughts * Conscientiousness	−0.111	0.080	−1.388	0.168	−0.270	0.048
Self-referential thoughts * Extraversion	0.051	0.079	0.646	0.520	−0.105	0.207
Self-referential thoughts	−0.077	0.024	−3.263	0.002	−0.123	−0.030

*Note*. YOE: years of education; PiL: purpose in life; CI, Confidence interval.

## Data Availability

The datasets generated and analyzed during the current study are available from the corresponding authors on reasonable request.
